# Proper nutrition and hydration are human rights: also and especially for older patients

**DOI:** 10.1007/s41999-023-00771-4

**Published:** 2023-03-31

**Authors:** Anne Marie Beck, Dorothee Volkert

**Affiliations:** 1 Dietetic and Nutritional Research Unit, EATEN, Copenhagen University Hospital-Herlev Gentofte, Borgmester Ib Juuls Vej 1, 2720 Herlev, Denmark; 2 Institute for Biomedicine of Aging, Friedrich-Alexander-Universität Erlangen-Nürnberg, Kobergerstraße 60, 90408 Nuremberg, Germany

It is well known among geriatricians that older people are at increased risk of malnutrition, and thus, malnutrition is widespread among older patients. According to a meta-analysis of prevalence data using MNA^®^, 22% of older hospital patients are malnourished and another 47% are at risk [1]. Also, the close relation between malnutrition and poor outcome is well documented and well known. Besides increased infections rates, length of hospital stay, duration of convalescence, and mortality risk, older people with malnutrition are predisposed to sarcopenia, frailty, and disability [2–5].

Just as frequent and at least as serious is the problem of dehydration, which in old and very old patients is usually caused by insufficient fluid intake (low-intake dehydration) [6]. Older persons are at increased risk of low fluid intake for various reasons. Due to inconsistent definitions, prevalence rates vary widely [7, 8]. Adverse health consequences occur even much faster than the consequences of malnutrition and, in addition to human suffering, constantly cause enormous costs in the health care system.

An explanation for the high prevalence of malnutrition and low-intake dehydration may be—besides the increased vulnerability of older people—low awareness of the problem and of preventive possibilities among health care professionals and the assumption that interventions to address these problems in older patients are not very effective. As an example, a survey among European medical schools has shown that the topic of malnutrition in older adults was only included as part of the medical students’ curricula in half of the participating institutions [9].

However, this is not what the evidence tells us. In the ESPEN guideline on clinical nutrition and hydration in geriatrics, a systematic literature search identified numerous studies and systematic reviews supporting the beneficial effects of interventions targeting malnutrition and dehydration [10].

**Fig. 1:**
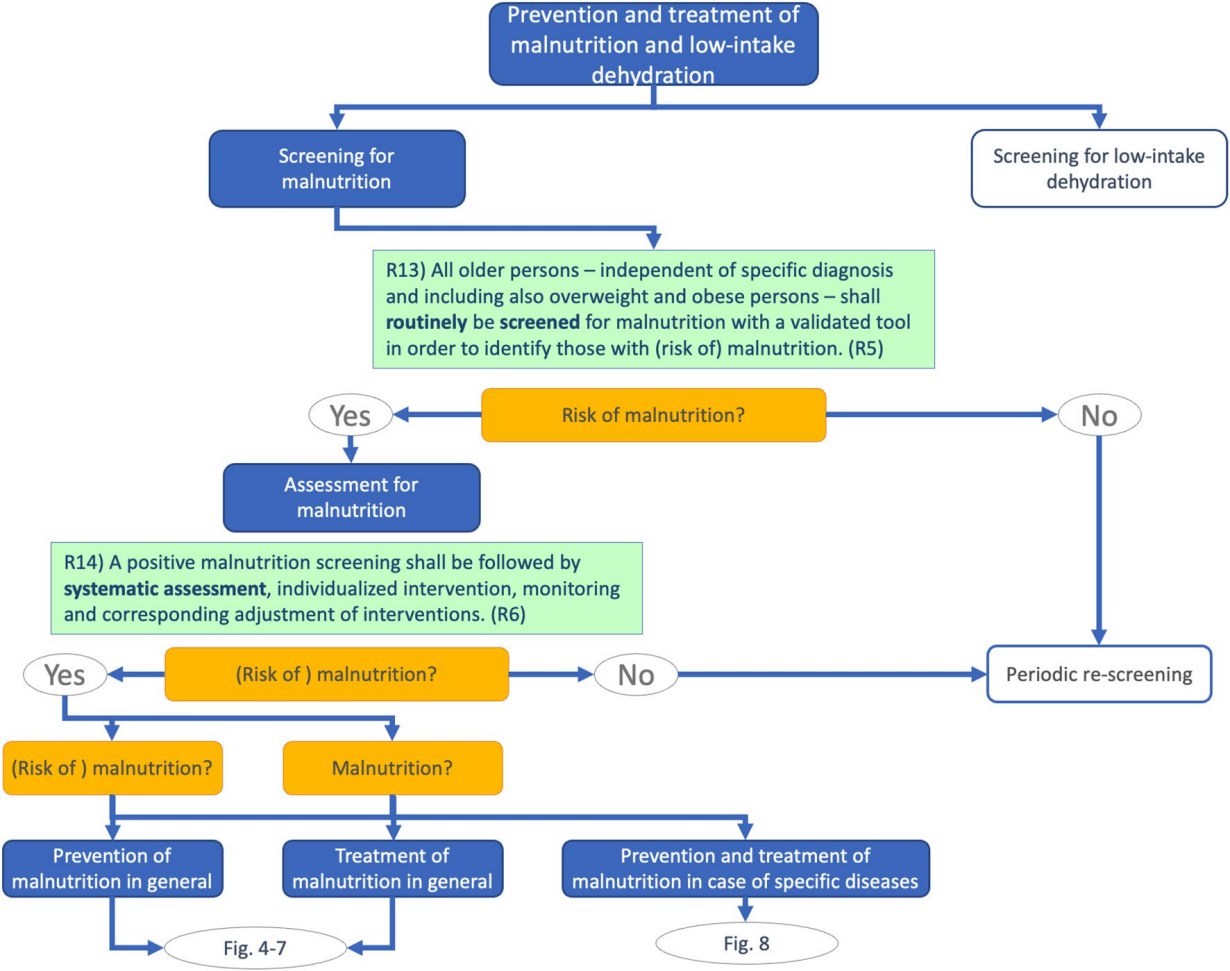
Prevention and treatment of malnutrition and low-intake dehydration—Screening for malnutrition [11]

**Fig. 2:**
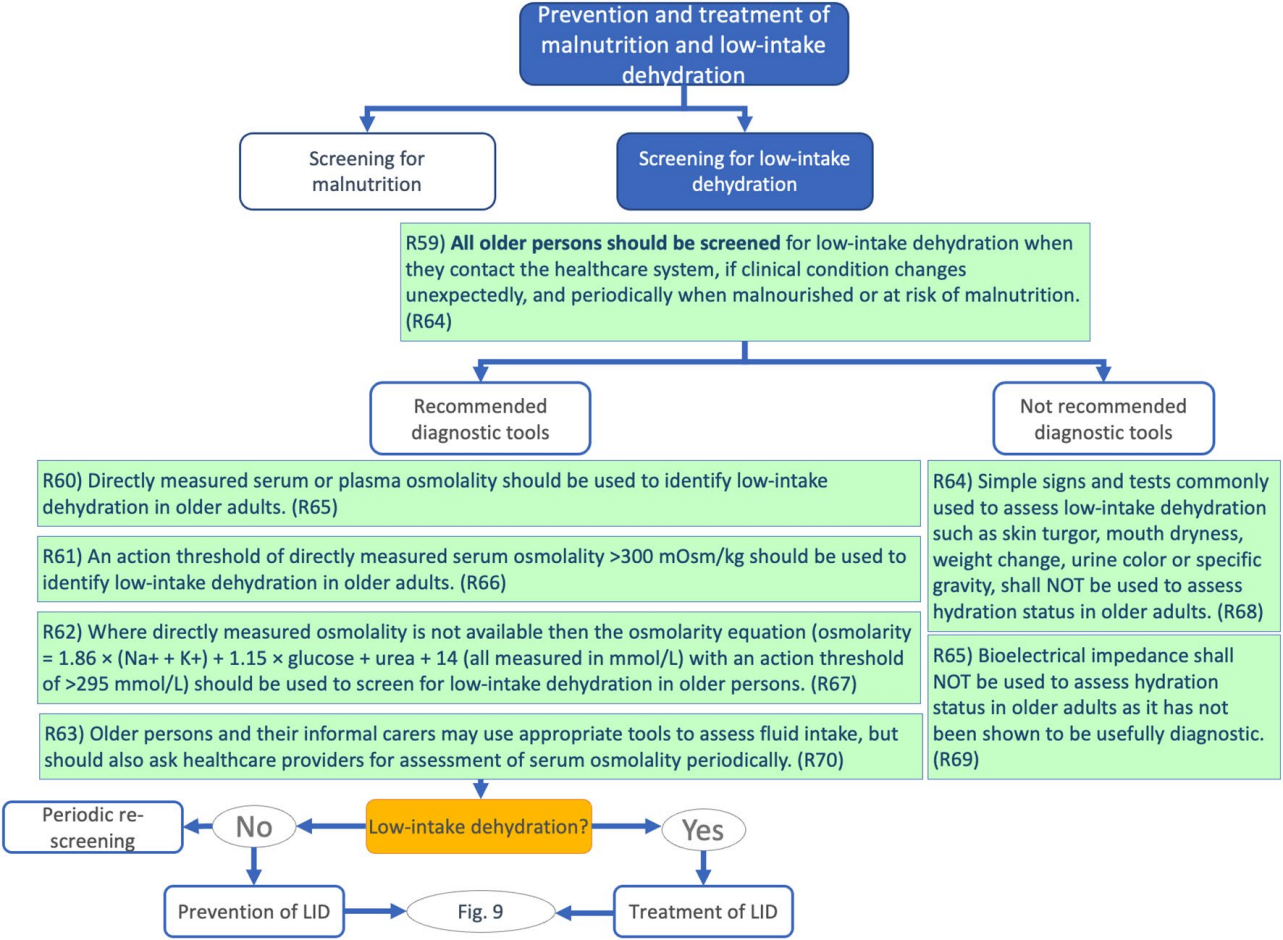
Prevention and treatment of malnutrition and low-intake dehydration — Screening for low-intake dehydration. *LID* low-intake dehydration [11]

Recently, this guideline was further developed and published in the form of a practical guideline [11], which is also available as smartphone app to make it suitable for everyday use. The main difference from the original guideline is the graphical arrangement of the recommendations, which are unchanged in content. This new, algorithmic arrangement follows the clinical process of patient care from screening and assessment to the various treatment options and is designed to facilitate application and implementation in practice. Figures 1 and 2 are intended to give an exemplary impression.

The recent leading-edge EFFORT study in medical inpatients at nutritional risk with its impressive results underlines the recommendations of the ESPEN guideline. Although adult medical inpatients of all ages were included, mean age of the participants was 76 years and only 17.5% were younger than 65 years. Individualized nutritional care by dietitians with protocol-guided interventions to achieve specific energy and protein intake goals resulted in a reduced incidence of adverse clinical events within 30 days, as well as lower 30-day mortality and significant improvements in functional outcomes and quality of life among the more than 1000 patients in the intervention group [12]. In a subgroup analysis of patients with aging-related vulnerability, defined by advanced age (80 + years), physical frailty or cognitive impairment, the effects were even more pronounced with a more than 50% reduction of the risk of 30-day mortality and in addition significantly reduced mortality at discharge and after 180 days [13].

Evidence is thus available and summarized in applicable recommendations. What is unfortunately still largely missing, however, is the actual implementation of these recommendations in clinical practice. This could be easily supported by incorporation of the guidelines into quality assurance frameworks and active guideline implementation strategies in geriatric institutions [2].

Now exactly 20 years ago, already, the Council of Europe in their resolution on food and nutritional care in hospitals emphasized access to a safe and healthy variety of food as a fundamental human right. In view of the beneficial effects of adequate nutrition care in hospitals on the recovery of patients and their quality of life, the implementation of recommendations in this regard was claimed [14].

In conclusion, proper nutrition and hydration are human rights also in older patients and there is no longer any excuse for not considering this.
